# Disability and masculinity in South African autosomatography

**DOI:** 10.4102/ajod.v3i1.85

**Published:** 2014-04-01

**Authors:** Ken J. Lipenga

**Affiliations:** 1 English Department, Chancellor College, University of Malawi, Malawi; 2 English Department, University of Stellenbosch, South Africa

## Abstract

This article examines the representation of disability by disabled black South African men as portrayed in two texts from the autosomatography genre, which encompasses first-person narratives of illness and disability. Drawing on extracts from Musa E. Zulu’s *The language of me* and William Zulu’s *Spring will come*, the article argues that physical disability affects heteronormative concepts of masculinity by altering the body, which is the primary referent for the construction and performance of hegemonic masculinity. In ableist contexts, the male disabled body may be accorded labels of asexuality. This article therefore reveals how male characters with disabilities reconstruct the male self by both reintegrating themselves within the dominant grid of masculinity and reformulating some of the tenets of hegemonic masculinity.

between these thighs i am a complete man
between these thighs an equal woman
i am
between these thighs i can impregnate
and between these thighs i can be
impregnated
Matoto (2010:148)

## Introduction

This article examines *The language of me* (2004) and *Spring will come* (2005) by Musa Zulu and William Zulu respectively, in terms of how the narrators negotiate their position in relation to the heteronormative model of hegemonic masculinity. The two texts fall into the genre of autosomatography, a subcategory of the autobiography genre focusing on the disabled or ill body. It is a genre that highlights ‘what it’s like to have or to *be*, to live in or *as*, a particular body – indeed, a body that is usually odd or anomalous’ (Couser 2009:2). This genre is particularly effective since, firstly, it is an expression of the ‘Nothing about us without us’ slogan that has been adopted by many disability activists, which asserts the need for people with disabilities to be part of any decisions made about them. In addition, as a sub-genre of autobiography, these texts are ‘sites of identity production [*as they*] both resist and produce cultural identities’ (Gilmore 1994:4). The memoirs examined here do precisely that, through an articulation of positions that simultaneously challenge hegemonic masculinities and create space for alternative masculinities.

Focusing on the human body can reveal its position in society as ‘[*a text*] in which we can read the ideological assumptions of the social system: [*as a text*] for understanding social institutions, social discourses and social forms’ (Meekosha 1998:172). The disabled body illustrates this function by highlighting its resistance to homogenous labels of the norm. As David Mitchell and Sharon Snyder (2000:6) observe, ‘it is the narrative of disability’s very unknowability that consolidates the need to tell a story about it’. To a certain extent, these texts address this need for narrative, particularly as a way of highlighting the intersection of physical disability with hegemonic masculinity.

One could relate these narratives to the findings of research conducted by Gershick and Miller (1997) on the strategies employed by disabled American men to articulate masculinity. The two scholars conclude that in negotiating masculinity, disabled men usually follow three patterns: reformulation, reliance, and rejection – of the values upheld by the dominant masculinity. This ‘Three “R” Framework’, as the authors call it, is helpful in reading the manner in which William Zulu and Musa Zulu negotiate their masculinity.

## The club of hegemonic masculinity

In their book, *Men’s health and illness*, Donald Sabo and David Frederick Gordon (1995:10) argue that ‘[*t*]here is no such thing as masculinity, there are only masculinities’. This statement is supposed to buttress the point that we cannot place all forms of male experience under one label. ‘At any given historical moment,’ they argue, ‘there are competing masculinities – some hegemonic, some marginalized, and some stigmatized – each with their respective structural, psychosocial, and cultural moorings’ (Sabo & Gordon 1995:10). However, this multiplicity of masculinities is partially informed by ‘a system of internal dominance in which a minority of men dominates the masses of men’ (Sabo & Gordon 1995:10). This dominant cadre forms what Bob Connell (1987) calls hegemonic masculinity.

One of the variables used to define masculinities is context – they can be read as being ‘society-specific’ (Uchendu 2008:3). We are thus able to talk of African masculinity. Such masculinity is defined not only by geography but also by attendant cultural factors extant in that physical space. Much as this might be the case, even within Africa there are different forms of masculinities (Ratele 2008b:25). In South Africa, for example, according to Kopano Ratele (2008b:25), ‘a heterosexual patriarchal capitalist masculinity is the hegemonic form of masculinity’. This form of masculinity is hugely sought after by most South African males of all races, and it is characterised by the values of aggressiveness, courage, strength, drive, ambition (career-orientedness) and self-reliance.

Unsurprisingly, hegemonic masculinity is often held by its adherents as the most proper form. Consequently, anyone who fails to evince the required character traits is considered not fully masculine. Most conceptions of hegemonic masculinity define and locate it in the male white body (Morrell 1998:608; Saint-Aubin 2005:31), making race one of the factors that often restricts belonging to this elite group. Apart from race, however, entrance into the fold – this club – of hegemonic masculinity could be denied on a number of other grounds, including ethnicity, class, sexual orientation or physical disability (Gershick & Miller 1997:456; Morrell 2007:16). It is this category of physical disability as a restricting factor that the article examines, particularly as it is portrayed in *The language of me* and *Spring will come*.

For most disability scholars, the term autobiography is not sufficient to categorise life writing texts by (or on behalf of) disabled subjects. The genre evokes a literary practice that lays emphasis on a certain kind of normalcy (Quackenbush 2008:23). Hence the turn by scholars such as G. Thomas Couser (2009:3) to formulate particular sub-genres capturing ‘disability [*as*] one of the pervasive topics of life writing’. Compared to the Global North, life writing texts on disability are not common in Africa. And even those that exist are not that well known, unless they are on (or by) particularly famous people. Most people are at least aware of Oscar Pistorius’s *Blade runner* (2009) and Natalie du Toit’s biography, *Tumble turn* (2006), and perhaps the Oprah Winfrey-narrated documentary *Emmanuel’s gift* (2005). But few people know of Esther Owuor’s *My Life as a paraplegic* (2000), Mongezi Ngidi’s *Black or white: ‘Does it matter’* (2005) or even of the Oscar-winning short documentary *Music by prudence* (2010). The latter group falls into the category of what Lorraine Adams (2002) calls the ‘nobody memoir’, life narratives by people who are not already well known prior to the publication of their life stories. These are all life narratives of people with disabilities in Africa, and they are amongst texts that engage in ‘the fundamental endeavour to destigmatize various anomalous bodily conditions’ (Couser 2009:6). The texts that are under discussion in this paper also fall into this category, since they were not penned by already famous personalities.

## Disability and hegemonic masculinity

Despite the widespread use of Connell’s (1987) model of hegemonic masculinity, I agree with Stephan F. Meischer and Lisa A. Lindsay (2003:6) who warn that ‘studying masculinity in African situations requires using [*the hegemonic masculinity model*] with caution’. This very important point realises the problematic nature of assuming the universality of a single model of hegemonic masculinity. This is even more pertinent when it comes to studying representations of black African males who are disabled. Interestingly enough, although hegemonic masculinity, as articulated by Carrigan, Connell and Lee (1985) is based on the white male body, most black African male communities often display valorisation of the same values identified in the hegemonic masculinity model (aggressiveness, independence, etc.). In South Africa, which forms the setting of *The language of me* and *Spring will come*, dominant models of masculinity have been affected by the history of the country:

Hegemonic masculinities in South Africa have both formed and been formed by the politics of racialisation. The apartheid state linked to a particular form of capitalist accumulation in South Africa provided both the conditions for the social construction of white male identities, and for the formation of masculinities linked to subordinated racialised groups. (Unterhalter 2000:162)

Capitalist accumulation is also drawn from traditional Zulu models of masculinity, where, for example, in the early 19th century the accumulation of cattle is important for a man’s reputation as an *umnumzana* [household head] (Hunter 2005:143). In apartheid (and post-apartheid) South Africa, this translates into the need for black men to find employment in order to support their families.

The problem arises from the way our ableist societies idealise particular bodies, and denigrate others. Lenore Manderson and Susan Peake (2005:233) argue that ‘[*b*]ecoming disabled for a man means to “cross the fence” and take on the stigmatizing constructs of the masculine body made feminine and soft’. Values of masculinity are imparted onto both male and female children by various socialising agents in their societies, including their parents, siblings and peers. The same society defines which bodies are to be valued. Physically disabled men’s sense of diminished masculinity therefore arises from the bodily valuation that is absorbed as one grows up.

## Spring will come

William Zulu’s *Spring will come* details the author’s experiences from childhood to the present. In the book, the author traces his Zulu (2005:1) ancestry back to 1879, ‘the year that effectively marked the end of the great Zulu empire and its illustrious kingship’. His disability is the result of two factors: spinal tuberculosis and a misplaced surgical operation in his teenage years, the result of which is paralysis in the lower half of his body.

*Spring will come*’s historical and cultural settings are crucial in forming some sense of the models of masculinity that the author draws on. The story spans from the late 1950s to 1994, the year of regime change in South Africa. The main events of the story are therefore set in the apartheid era. This setting reflects the narrator’s dependence on masculinity models constructed from interlocking sources. On the one hand, the author’s pride in his culture reflects the influence of traditional Zulu models of masculinity. In the early parts of the text, his symbol in this regard is Mangosuthu Buthelezi, leader of the Inkatha Freedom Party (IFP) and a prominent royal personality amongst the Zulu. The narrator writes of him in this fashion:

The Chief stood for my Zulu people far away in the land of the Zulus, where I belonged. Looking at him, regaled in his traditional skins with shield and assegai, his visionary eyes staring directly at the world, I would feel my blood stir with pride. (Zulu 2005:81)

On the other hand, living in the poor Emondlo township as part of the oppressed, largely impoverished black race, Zulu is driven by the need to assert his masculinity through the attainment of a stable source of income and the accumulation of material wealth. As a disabled black man in this society, the pressure to prove his masculinity is even more pressing.

The subject of masculinity emerges at several points in Zulu’s narrative, mainly associated with a deeply entrenched belief in his sexual undesirability. For the author, the idea of masculinity is strongly tied to perceptions and performances of sexuality. His conservative Zulu community further emphasises the link between masculinity and sexuality through its valorisation of men who have sired children (Hunter 2006:99). In her introduction to intersectionality in the family, Patricia Hill Collins (1998:63) argues that various systems of oppression usually work ‘mutually’ to ‘construct one another,’ rather than existing independently. Her insight is here evoked to assist in understanding the intersection of masculinity and disability, as illustrated in *Spring will come*, where the narrator struggles to recognise his validity as a man. Not surprisingly, Zulu’s conviction about the unattractiveness of his body takes root whilst he is admitted to hospital, recovering from an operation and undergoing physical therapy. As a space purposefully designed to house those who are either deemed infirm or afflicted with disease, the hospital ward creates – in Zulu – a sense of ‘weakness.’ Zulu’s sense of diminished masculinity is further emphasised by a nickname he is given by one of the medical orderlies, who teasingly calls him ‘Bachelor-boy’. This name arouses mixed feelings in him:

Although I understood it as well meant by Malume, [*my nickname*] made me conscious of my missing relations with the opposite sex. I would quickly dismiss such thoughts by reminding myself that I was paralysed now and that any relationship was out of the question, yet I still enjoyed watching the young women who came to see the other patients during visiting hours. (Zulu 2005:70)

This passage reveals the narrator’s belief that his changed body renders him an unsuitable sexual partner. Zulu’s long held assumptions regarding the propriety of romantic relationships and (it appears) of sexual encounter present an example of ‘how deeply some men with physical disabilities internalise hegemonic standards of desirability and sexuality which make them complicit in their own domination’ (Gershick 1998:199). Even after he leaves the hospital, he does not dare engage the opposite sex in any intimate relationships for fear of rejection.

An effect which further destabilises the author’s sense of his masculinity in his youth is the sense of infantilisation that emanates from diminished independence brought about by loss of functionality. The ‘Bachelor-boy’ tag is a reminder that he might never assume the ultimate status as a *man*, but is forever condemned to be a ‘boy’ (which incidentally parallels the way the term ‘boy’ was employed by the ruling white minority in denigratory address to black men). Subsequent events in the narrative overturn this assumption, but it is important to note the narrator’s assumption of having reverted to childhood as a result of the disability.

The author’s thoughts regarding diminished masculinity and infantilisation reveal an unconscious subscription to hegemonic masculinity, where the ideal masculine body is also the able-bodied one. This leads to a sense of isolation, not only from society in general but also from the category of ‘desirable’ men. The statement, ‘I was paralysed now and … any relationship was out of the question’ confirms this conviction. It is further entrenched by the weakening of his Christian faith:

In his senseless way god had disabled my body and left in it a living heart that yearned, desperately, to love and be loved. If he could listen to my heart, surely he had heard it many times thumping excitedly and silently calling out to a lovely dame, while my eyes drank in her ripe African beauty, wishing I could propose. (Zulu 2005:208)

This is a passage that effectively illustrates the overlapping of disability with religion and masculinity. In the text, religion (in the form of the Christian God as well as ancestral worship and beliefs in witchcraft) is constantly evoked as a way of musing on possible causes of the author’s disability. In the same passage, however, the author touches on the need for love. His expression of this desire evinces a split self. The narrator regards his body as an external part of him, which nevertheless houses a highly sensitive being, a part that is crucial in articulating modes of masculinity that depart form the hegemonic forms. It is this physical part of him – he is convinced – that denies him emotional fulfilment.

Although the author initially deems himself unsuitable for feminine company, these feelings are born out of an ableist undervaluing of the disabled body. He eventually reads his persistent desire for a woman as an expression of heterosexual masculinity, and realises that the loss of feeling in his legs does not entail a loss of his masculinity. The object of his desire, whom he eventually marries, is lovingly described as a woman:

in her mid-twenties, judging by her full, curvaceous figure, [*with*] a round, light complexioned face with large, clear eyes and a full mouth that pouted tantalisingly, as if ready for a kiss. (Zulu 2005:292)

There are two important points that emerge from this description. In the first place, it betrays the author’s desire for what can as well be called a hegemonic femininity. Secondly, that he is drawn to her due to her physicality indicates a turning point not only in the narrative, but also in his sense of self-worth, reflected in the narrator’s admission that ‘as [*he*] gazed at her, [*he*] forgot [*his*] self-consciousness and [*his*] disability’ (Zulu 2005:292). In this reversal is a clear illustration of the re-establishing of a sense of selfhood. The affection he shares with his wife removes his impression of his body as an impediment to the attainment of a love relationship. Remarkably, he describes his wedding day as ‘the day I truly became a man’ (*ibid*:306) – suggesting a transcendence of both the bachelorhood and boyhood of his aforementioned nickname whilst also validating the idea that his disability does not deny him his masculinity. This statement also reflects the community’s valuation of a man when he finds a wife. In line with Gershick and Miller’s framework, William Zulu’s position also betrays a *reliance* on the hegemonic, heteronormative masculinity model at this point. In this model, the performance of masculinity involves a stage when the male must conquer the female other, particularly through wooing (Shuttleworth 2004:170). As a black man in apartheid South Africa, his ability to wed (especially with the bride price of seven cows) is a display of masculine economic muscle, since even in the democratic era, ‘wedlock continues to remain outside the scope of most young men’s financial capacity’ (Hunter 2005:149).

In *Spring will come*, the articulation of masculinity is not only done through the attainment of matrimony. For William Zulu, his masculinity is expressed in other ways, some of them material. For example, after the fateful operation, Zulu’s greatest worry concerns the resulting limited mobility. He sees his reliance on a wheelchair as a marker of loss of independence. The need to regain this independence convinces him that ‘[*t*]he way out of [*his*] loneliness and isolation was to buy a car and drive away to Jo’burg’ (Zulu 2005:283). The car would free him from the immobility of disability. Significantly, when he eventually obtains the vehicle, he feels ‘in control, driving my own car and somehow feeling master of my own destiny’ (*ibid*:286). The vehicle restores a sense of autonomy that he felt missing from his life (even though he never moves to Johannesburg). His control of the car thus mirrors a re-attainment of control of his own life, and he achieves independence, which is a central requirement of hegemonic masculinity.

Zulu’s success as an artist also paves way for him to assert his masculinity in two ways. The first is through the improvement in his economic position. With the proceeds from sales of his artwork he has a house built according to his own plans, at the completion of which he feels ‘a man amongst men’ (Zulu 2005:187). This fits directly into the value of self-reliance upheld in hegemonic masculinity. More importantly, constructing a house is an expression of a man’s ability to support a family. The ideal male is supposed to be career-oriented and achieve economic independence, usually in order to fulfil what Lisa Lindsay (1999:784) calls the ‘breadwinner ideal’. This is an ideal that is also emphasised in Zulu culture (Waetjen & Maré 2001:203). For William Zulu, this is particularly significant given that in most African communities, ‘people with disabilities are most often severely disadvantaged on the employment market’ (Ingstad & Eide 2011:5). The ability to gain economic independence therefore emerges as a significant victory when the odds of finding gainful employment are stacked against not only the disabled male, but the majority of black men in South Africa (Ratele 2008a:529).

The narrator’s creativity also opens up another avenue for articulating his masculinity. This is a personalised model in which Zulu emphasises his masculinity through various media of expression, including prose and verse, as well as linocut prints. As he hones his artistic skills, he appreciates more and more his mental faculties over his bodily existence:

In my isolation as an artist, I understood myself as being made up of two personalities. The first was a creative individual with a lively mind, a sense of fun and a spirit of independence, while the second was a prisoner trapped in an ailing body, which felt like a millstone, dragging me down into an abyss of hopelessness. (Zulu 2005:146)

The narrator’s words evoke Kristin Lindgren’s (2004:155) observation that autosomatographical writings often ‘represent subjectivity as split or doubled, as comprising both self and other’. The sense of split identity is informative of why William Zulu prefers to emphasise the mind over the body. As an artist, perhaps it can be easily understood why he values his creative faculties a great deal. His linocut prints feature black South Africans, usually in various scenes of toil, poverty or some other form of suffering. Through them, in acknowledging the plight of black people in South Africa, he challenges the idea of the disabled writer as a ‘singular subject’ (Mitchell 2000:311). On the contrary, as the author-artist points out in an interview, he sees himself as ‘a social and political commentator through [*his*] art’ (Newman 2011). The writer-artist’s creativity therefore permits him access to other spaces beyond his immediate confines. The freedom and creativity achieved through his artworks opens up a world beyond his home, permitting him to express himself not as a solitary disabled figure, but rather as a citizen of the nation, identifying with other people of his race. This agency, afforded by Zulu’s creativity, emerges as a challenge to the limited mobility that his disability represents. The fact that his artworks later gain him access to international audiences further strengthens their enabling potential.

## The language of me

Musa Zulu’s *The language of me* is a memoir in which he traces phases of his life before and after the car accident which leaves him paralysed from the waist down. The book details the author’s emotional relationship to his new body, highlighting the way he struggles to reject older, ableist ways of regarding disability, and eventually showcases avenues that he discovers which enable him to appreciate the joys of life.

*The language of me* is strictly speaking more of a memoir than an autobiography. This is because instead of tracing a broad range of events in the author’s life, it focuses of a limited, focused set of experiences. As Zulu (2004:x) puts it: ‘[*the*] book is a record of my life between 1995 and 2001, the years of my journey through the light and shadow of disability’. This time-frame sets the book in the early years of post-apartheid South Africa, making the context different from that of *Spring will come*. Another key difference is Musa Zulu’s economic situation, which is noticeably better than William Zulu’s. At the time of the accident, he has sufficient medical aid to be treated at ‘a private hospital of world-class standard’ (*ibid*:22), unlike the faith healers, *inyangas, sangomas* and government-run hospitals that William Zulu has to contend with.

Despite these differences, the influences on Musa Zulu’s ideas of masculinity are not very different from those of William Zulu. He too is influenced by cultural models of masculinity. His Zulu ancestry is constantly evoked in this regard. He describes himself as ‘a proud descendant of the house of Zulu’ (Zulu 2004:105). However, Musa Zulu’s difference emerges in the fact that he prefers to emphasise the undervalued traits of Zulu manhood, ‘never [*having*] been happy with the stereotypical image that has come to be accorded to [*the Zulu*] people – “aggressive”, “war-mongers”, “uncultured”, and the like’ (*ibid*:105). In direct contrast to the warrior image so ardently admired by William Zulu, Musa Zulu prefers to distance himself ‘from all the usual clichéd associations – the animal skins, drums, shields and spears’ preferring instead to celebrate ‘the beauty, wisdom and maturity of [*his*] culture’ (*ibid*:105). His is therefore a conscious attempt to deny the violence that is normally associated with Zulu masculinity. Musa Zulu (2004) is also influenced by the township culture of masculinity, where he realises:

from an early age that part of ensuring a man’s ‘survival’ in the masculine social jungle, and gaining the respect of other boys, lay with the art of attracting women. (p. 96)

As a result, prior to his disablement, he has ‘a string of relationships and encounters’ (Zulu 2004:95) before settling down. Although the politics of the ‘new’ South Africa does not affect the author’s notions of masculinity, it nevertheless informs the drive that he has to play his part in transforming the lives of ‘the marginalised black population, whose lives had been badly disrupted by the years of political oppression’ (*ibid*:15). This motivation later translates into the formation of a Support Group for the Disabled, through which the narrator and his colleagues engage in outreach to other disabled people in hospitals.

For both authors, mobility is essential not merely for gaining access to particular spaces, but also as an expression of choice. Reflecting on his past in the text, Musa Zulu (2004:36) observes: ‘I had always been a fast mover – in all senses of the term – delighting in speed, in a race, in outdoing the competition’. Mobility is presented in literal terms, referring to movement of his legs, but also metaphorically evoked to represent movement in his career. When he is paralysed, however, he feels that life as he knows it has come to an end:

Young as I was, I had already accomplished so much in my life and was looking forward to achieving so much more. I was at the peak of my potential, in the process of spreading my wings for still greater heights. My goal was to vault into the skies and shine up there with all the other stars. It was a crushing blow to realise that those big ambitions had died in the week along with the person I used to be … I used to cry a lot during those early days. (*ibid*:21)

One of the most striking aspects of Musa Zulu’s language is indeed how personal it sometimes becomes (as the title indicates). His pains and losses are expressed in a metaphorically rich and impassioned voice that effectively communicates the experiential from this individual’s unique perspective. In the passage above, the emotion is captured primarily in the emphasis on youth and accomplishment, expressed in the language of upward mobility. Similar to William Zulu’s language in the early part of *Spring will come*, Musa Zulu’s language here illustrates the author’s ableist conviction that disability entails failure in every other enterprise in life. Like William Zulu, Musa Zulu realises the blow to his masculinity whilst in the hospital space. As a paralysed patient he requires assistance in several respects:

One of the most devastating things about paralysis is the way it impacts on normal bodily functions. Because I could no longer urinate in the normal way, I had to use a catheter to empty my bladder… I hated that bloody catheter. It became my worst enemy. I found it completely humiliating to have to fiddle with myself, poking about in my penis, trying to insert the tube into the right channel. It was like puncturing the very essence of your manhood, tampering with the core of you. (Zulu 2004:23)

One unique feature about the autobiographical mode is how it often uses the first person narrative form to draw the reader into intimate spaces. As a result, the reader easily shares emotional experiences depicted by the author. As the passage above clearly indicates, in his conception of self, the disability injures ‘the very essence of [*his*] manhood […] the core of [*him*]’ (Zulu 2004:23). This indicates another aspect of the self that is directly related to the body. It is a gendered self that has been damaged here, the male self. The penis is the bodily indicator of that self, and the fact that that part is being ‘tampered’ with is another indicator that his essence, the very ‘core’ of him, has been exposed and disabled. For the penis to lose some of its function represents a huge blow to a male human being. As ‘the core of [*him*]’ the penis is not just another limb, but, to the narrator, the one limb that indicates his masculinity. As the anthropologist Robert F. Murphy (1987:96) so bluntly put it in *The body silent*, ‘being a man does not mean just having a penis – it means having a sexually useful one. Anything less than that is indeed a kind of castration’. For the male author, the penis represents not only manhood, but actually represents his (male) identity as a person. This is the case in most forms of hegemonic masculinity. For a man to move to a position where the penis no longer figures strongly in his sense of himself as a man requires an adoption of alternative models of masculinity, which are usually in the minority, compared to any form of hegemonic masculinity that may be in vogue in his cultural milieu.

The passage on the affected penile function cited above reveals how Musa Zulu initially views his disability as damaging to his sense of selfhood by reflecting on his changed body. This is seen not only in both the loss of mobility and the exposure of and apparent diminished functionality in a vital bodily part of his male self. The catheter – an appendage – is connected to the penis as the ‘master-signifier’ of masculinity (Connell 2001:37), an indication not only of the damaged urinary function, but a blow to the man’s self-esteem. This is echoed on the various occasions in the narrative when the author mentions loss of erectile function.

Given the centrality of the penis to a man’s self-image, encounters with the opposite sex (assuming a heterosexual inclination, as is evident in Musa Zulu’s case), especially intimate ones, serve either to emphasise diminished masculinity or to reinforce the existing sense of one’s masculinity. In one study, Russell P. Shuttleworth (2004:169– 170) observes that for disabled men, ‘confronting the dilemma of how to be masculine […] is felt most acutely during their interpersonal attempts to establish sexual intimacy with others’. Such encounters create anxiety due to the expectation in the men of a particular kind and ‘level’ of performance. Musa Zulu narrates an unfortunate experience he had with his girlfriend that highlights this phenomenon. As an indication of his having a diminished sense of self-worth, he convinces himself that it would not be fair to continue his relationship with his girlfriend. He feels he will be a burden to her. However, part of the reason underlying Musa Zulu’s decision is his anxiety concerning his virility. He explains the reasons behind his decision:

It was difficult for her, because I was no longer the man I used to be; it was not just my ability to walk that was lost, but a lot of other things. Disability steals away your sexual performance, and with that, your sense of confidence and control. […] I also remember the first night we spent together after I had left the hospital – she said she wanted to be with me. I could not get an erection and when I attempted masturbation to stimulate my penis, it only triggered my bladder and I wet the bed. My God – how it blew me apart! I could have killed myself right there and then. (Zulu 2004:31)

This passage hardly needs any elucidation, as the narrator’s anguish is clearly and painfully communicated. Expressing the belief that he is ‘no longer the man [*he*] used to be’ is perhaps the most straightforward illustration of Musa Zulu’s sense of diminished masculinity. Furthermore, the passage carries a hint of nostalgia for a past when he had been a more ‘complete man’, who, in his mind at least, was indeed deserving of love and affection. Wanting to dismiss his partner is therefore a statement to the effect that he is not only less of a man, but also less of a person, since, it is implied, only a ‘complete’ person is worthy of love. This passage moves the reader to a realisation of the agony that the narrator goes through. The narrator invites the reader into a scene of extreme intimacy and privacy, and then lays bare the humiliating, embarrassing and to him devastating outcome of that encounter. Such honesty has the effect of approximating the anguish that the writer feels, further revealing what it means for this particular individual to be disabled, and what this does to his sense of sexual masculinity – what one might term his sexual pride. Injury to the spinal cord usually affects a man’s virility because it ‘commonly produce[*s*] some degree of impotence or sexual malfunction’ (Murphy 1987:95). This creates a sense of ‘symbolic castration’ (Murphy 1987:96) in men, due to the apparent loss of function in the male member. Entry into Musa Zulu’s thoughts reveals a loss of control over that ‘master-signifier’ of his male being. The fact that he cannot control his penis, in the presence of a woman, seems to confirm the label of a ‘non-man’ that his disability threatens to confer upon him.

One of the most persistent ableist myths about disability is the attribution of asexuality, sexual deviance, or hypersexuality to the disabled body (Cohen-Rottenberg 2012; Siebers 2008:138; Shakespeare 1999:55). As Musa Zulu (2004:109) laments in the memoir, ‘so many disabled men find it so difficult to establish relationships with women – society alienates them from their manhood, since it defines them as being less of men’. His book is therefore an illustration of how he confronts some of those misconceptions in his society.

Similar to *Spring will come*, mobility is evoked in *The language of me* to describe Musa Zulu’s need for independence and control of his own life. In both texts, the loss of mobility threatens the man’s membership to the club of hegemonic masculinity since it jeopardises the ideal of independence. For Musa Zulu, the theme of mobility also finds its way into the very expressions that he employs. For instance, he speaks of wishing to ‘[*spread his*] wings,’ soaring to the ‘heights’ and ‘vault[*ing*] into the skies’ in figurative reference to his career dreams. However, mobility in the literal sense is also powerfully presented in the text, particularly with the narrator’s description of his first car, which he was driving at the time of the accident. Afterwards, his fear is that he ‘was doomed to a wheelchair’s pace of locomotion forever’ (Zulu 2004:24). Cars are central to the narrator, not just for their usefulness in transportation, but as representations of (largely masculine) achievement. Consider the following passage, where he describes the vehicle:

When I first learnt I was paralysed, I was terrified by the thought that my driving days were over. My family showed me photographs of my beloved Golf, which I had been driving at the time of the crash. I was horrified by the wreckage I saw and completely devastated by the thought that it had been my first and last car. I had loved that Golf like a part of me. I bought it on the 30th of August 1994 and the very next day it emerged from a Car Audio Shop, equipped with an uncompromising sound system. Off we went together – a marriage made in *boys’* heaven. I cared for that car with absolute dedication, kept it polished, vacuumed and serviced – in mint condition. The two of us had a wonderful relationship that was tragically terminated after only nine months by the collision with the fateful brick wall. We both suffered the heavy blows of impact and my baby was towed away to a scrapyard while I was being wheeled into the ICU – two lives forever separated. (*ibid*:24, [my emphasis])

The vehicle is here described in terms that almost define it as a human being, with whom the narrator has an amazing – though curtailed – relationship. However, the car, together with the sound accessories fitted in, reveal a particularly masculine pleasure, the loss of which Zulu later bemoans. Couser (1997) observes that:

the need to use a wheelchair literally lowers a person’s stature (and implicitly status), and the apparent uselessness of the lower body implies a lack of potency, sexual and otherwise. (p. 184)

It is not just mobility that is under threat, but rather a bodily agency that is closely related to masculinity.

Like the title suggests, Musa Zulu’s language invokes deeper readings to the narrative. His choice of gendered images and metaphors invites additional perspectives for examining the way the narrator relates to this moment of disablement. For instance, Zulu’s description of his feelings towards his vehicle evokes readings which highlight a series of interrelated themes, including masculinity, femininity and rebirth. His relationship with the car is described as ‘a marriage made in boys’ heaven,’ effectively evoking an air of masculine indulgence. However, the ‘marriage’ with his ‘baby’ is ‘terminated only after nine months’. This image replaces the images of masculine pleasure with those that hint at maternity, foreshadowing the rebirth that is to occur after he has been ‘wheeled to the ICU,’ through reformulation of masculinity later in the text. This rebirth is further captured in the fact that upon leaving the ICU, Zulu is heavily dependent on others, before finding his ‘feet’ again – just like a ‘baby’. These gendered images further lay emphasis on the subject of diminished masculinity discussed in the text. The metaphors of termination and rebirth rendered through these images subtly capture the process of the disintegration of one model of masculinity, and its eventual replacement by another. This sense of renewal is captured in the narrator’s belief that the ‘[*p*]aralysis has cast a little more magic on me and offered me the chance for a new beginning’ (Zulu 2004:55).

Like William Zulu, Musa Zulu’s re-assertion of masculinity draws both on the hegemonic model as well as a personal one. The subject of mobility remains central in his assertion of masculinity, as seen when Musa Zulu (2004) has the chance to drive a car with hand controls:

When I drove that car, it was like heaven had opened its doors for me to come in and rest in peace. I knew then that it was completely possible for me to drive, despite my paralysis, and the new dream I started to embrace right then and there was to get my own wheels and drive myself wherever I wanted to go. (p. 25)

Once again, the emphasis on driving finds its way into his language. The rediscovery of driving in this context reconnects him to his earlier (masculine) love for cars. After the accident, Musa Zulu leaves the hospital with ‘only one mission in [*his*] mind: to recover, whatever it took, get back in the driver’s seat of [*his*] life again’ (Zulu 2004:27). The imagery of ‘rest[*ing*] in peace’ in this context signifies its opposite connotation – it emphasises the calm, renewed conviction the author has of becoming successful. Driving thus becomes a metaphor for regaining control of his life.

The title of Musa Zulu’s memoir has additional significance in relation to the way he regards the intersection between masculinity and creativity. In contrast to the aggression emphasised in hegemonic masculinities, Musa Zulu (2004:90) reflects the belief that ‘a man’s language, if articulated well and creatively expressed, can paint the colours of his core and reveal the essence of the man in him’. The creative side of men, therefore, is just as capable of expressing their masculinity as their actions are. Musa Zulu’s prose, poetry and sketches are a manifestation of his conviction that ‘the core of a man is expressed not through his brute strength, but through his tender and creative side’ *(ibid*:100). It is therefore through the creative mode that the narrator articulates a personal masculinity model. His writings and drawings are acts of enablement not only affording him a voice, but also in permitting him a unique articulation of his masculine self.

## Negotiating paths of masculinity

As the discussions above illustrate, the authors of *Spring will come* and *The language of me* engage in a negotiation of masculinity that simultaneously relies on and reformulates some aspects of hegemonic masculinity. What emerges is not necessarily a novel model of masculinity, but rather a position that re-asserts the subject’s belonging to the elite club, whilst also emphasising some normally marginal aspects of hegemonic masculinities. The ableist rejection of masculinity in connection to the disabled body is therefore on the limited grounds of the ideal masculine body, which hardly even exists. This entails that the denial of the disabled body within the realm of hegemonic masculinities is more a feature of ableist attitudes than anything else.

One important feature of the texts – besides the stories contained therein – is the importance of the actual mode of expression that is the written memoir. If we shift focus to the act of narrating the story, the texts can be identified as acts of enablement, through their according of agency in giving the author a voice. The life writing genre permits the speaker to engage directly with the public, and potentially change stereotyped views held by that public:

Intentionally or not, and whether positively affirmed or contested by our audience, our autobiographical accounts become entwined in struggles about justice. We use these accounts to hold up to public scrutiny the values informing our lives and those of other protagonists. In doing so, we appeal for recognition of individual and collective identities. (Coullie *et al*. 2006:2)

From this statement, the genre of autosomatography can destabilise misconceptions that the ableist public may hold about disability and masculinity. That is part of the intention of the writers. In the introduction to his book, for instance, Musa Zulu (2004:x) points out that his writing is an attempt ‘to show the world that “disability” is not only a story to tell to others, but also a site of talents that deserve to be uncovered and exhibited’. Through their acts of creativity, these men discover cracks in the boundaries of hegemonic masculinity, and invent ways of simultaneously aligning with dominant models of masculinity and asserting their own personalised masculinities.

## Conclusion

In ending the discussion, I wish to briefly reflect on the strategies of these two authors in re-negotiating their masculine selves. Gershick and Miller’s (1997) study of disabled American men yields some similarities to the strategies adopted by Musa Zulu and William Zulu. From the evidence given above, both narrators do not reject dominant models of masculinity, but rather engage in both reliance and reformulation of such models. As indicated above, various behavioural traits and preferences indicate a reliance on certain hegemonic masculinity ideals. Reformulation entails a definition of masculinity along new lines, previously not emphasised. This is evident in both narrators’ emphasis on their creativity. However, the two authors also reposition themselves within the dominant frame of masculinity, through their emphasis on one key aspect of masculinity – independence. This is expressed through their need for (and acquisition of) mobility, achieved through their vehicles. And like most males, they also take pride in economic independence. However, the two texts analysed in this essay feature men with similar disabilities. It would therefore be interesting, for future research, to investigate the manner in which other disabling conditions can affect perceptions (and constructions) of masculinity.

Much as Gershick and Miller’s study helpfully categorises common strategies of disabled men with regard to masculinity, one must note the limitation that the study has in the sense of having a limited sample that comprises only American men with disabilities. We must allow for the effect of socio-cultural factors in shaping masculinities particular to certain locations. In the present study, the South African historical and political context has a bearing on the way the authors see their place in society. Similarly, their Zulu cultural background is a factor that cannot be ignored in the attempt to determine influences on their ideas of masculinity. Paying heed to these factors not only allows for a better informed analysis of masculinity, but also of disability in this context.
